# 

**Published:** 1871-08

**Authors:** 


### INDEX.

### A

Abbott, F. W., M. D., The Intraocular serous membrane and the Nature of Glaucoma. By Dr. A. Sichel. Translated from Les Annales d'Oculistique ..... 161
" " " On The Recent Advances in the Theory of Vision. By H. Helmholtz. Translated from Les Annales d'Oculistique, 209, 245, 295 346, 376, 416, 474
Abcess of the Temporal Region, from Gunshot Wound. Extraction of Ball. By J. W. Southworth, M. D. ..... 229
Abstract of the proceedings of the Buffalo Medical Association ..... 10, 94, 336, 460
Abstract of Remarks on Diseases of the Ear and their Treatment. Before the Rochester City Medical Society. By Charles E. Rider, M. D. ..... 89
Acute Rheumatism. On Sesquichloride of Iron as a Prophylactic of ..... 134
Albany County Medical Society. Resolutions of in Relation to Abortion ..... 143
Albany County Medical Society, Semi-Annual Meeting. Reported by J. S. Bailey, M. D. 470
Albany County Medical Society Proceedings of Semi-Monthly Meetings. Reported by James S. Bailey, M. D. 201, 241, 281, 331
Albany Hospital ..... 182
Albuminuria in Pregnancy and its relation to Puerperal Convulsions. By Geo. T. Campbell, M. D. ..... 405
Alumni Association of Buffalo Medical College ..... 153
American Medical Association ..... 238, 361, 398
An Abortionist's Diploma ..... 75
Aneurismal Tumor following penetrating Wound of the Shoulder. Operation for ligation of Right Sub-Clavian Artery. Report of Case By C. C. F. Gay, M.D. 424
Appointment ..... 75
Apyretic Malarial Menorrhagia. By J. W. Southworth, M. D. ..... 174
Arterial Transfusion of Blood ..... 232
Atlantic Monthly ..... 402

### B

Bailey James S., M D., Report of Semi-Annual-Meeting of Albany County Medical Society ..... 470
" " " Report of Semi-Monthly Meeting of Albany County Medical Society, 201, 241, 381, 331
Bartlett, F. W., M. D., Diptheritic Croup and its Treatment ..... 321
Books and Pamphlets received 40, 8[illegible], 118 159, 200, 240, 319, 364, 402, 444, 484
Bradnack, F., M. D., A New Method of Treating Chronic Alcoholism ..... 44
" " " Periodical Headache A New Method of Curative Treatment. 220
Brecht, F. E. L., Reports of Clinical Remarks on Surgical Cases in the Hospital of the Sisters of Charity, By Prof J. F. Miner, M. D. 99, 100, 101, 127 128, 129, 177, 179, 180
Bromide of Potassium. On the Physiological Action of ..... 135
" " " Some of the ill effects of ..... 3[illegible]8
Bronchitis, Capillary in Children, Treatment by Warm Vapor ..... 19
Brush, Edward N., Reports of Clinical Remarks in the Hospital of the Sisters of Charity. By Prof. Th. F. Rochester, M. D. ..... 121, 264, 266
Buffalo Medical Association. Abstract of Proceedings ..... 10, 94, 336, 460

### C

Calabar Bean in "Spotted Fever" ..... 361
Campbell, Geo. T., M. D., Albuminuria in Pregnancy and its Relation to Puerperal Convulsions ..... 405
Capillary Bronchitis in Children, Treatment by Warm Vapor ..... 19
Carbolized Catgut Ligatures, The use of. 303
Carbolic acid as a local Anæsthetic. The application of ..... 63
Cerebro-Spinal Meningitis ..... 301
" " " Appearance in Buffalo ..... 30[illegible]
" " " In Canada ..... 355
" " " or "Spotted Fever" ..... 359
" " " Subsidence of, as an Epidemic in Buffalo ..... 481
Cervix Uteri, Ulcers of ..... 67
Chicago Medical Examiner ..... 199
Chloralum ..... 32
Chloroform, Deaths from ..... 239
Cholera Contagion, Prof. Crocq of Brussels on ..... 239
Chronic Alcoholism. A new method of treating, by Chloral Hydrate and Bromide of Potassium. By F. Bradnack, M D. ..... 44
Clinical Experiences in Private Practice. By Z. C. McElroy, M. D ..... 365
Clinical Remarks upon Surgical Cases in the Buffalo Hospital of the Sisters of Charity. By Prof. J. F. Miner, M. D. Reported by F. E L. Brecht. On Enchondroma ..... 99

Clinical Remarks on Cataract 100
" " " Inverted Toe-Nails 100
" " " Catarrhal Conjunctivitis 101
" " " Caries of the Bones of the Foot 127
" " " Concussion of the Brain 128
" " " Spinal Curvature 129
" " " Strabismus 177
" " " Non-Union of Fracture 179
" " " Syphilis 180
" " by Prof. Th. F. Rochester in the Buffalo Hospital of the Sisters of Charity. Reported by Edward N. Brush. On Erysipelas 131
" " " Diabetes 264
" " " Delirium Tremens 266
College of Veterinary Surgeons 40

CORRESPONDENCE.

Cerebro-Spinal Meningitis in Canada. 355
Clinics in Bellevue. C. H. Richmond, M. D. 112
Genesee County Medical Society 231
Homeopathy and Regular Medicine H. R. Hopkins, M. D. 15
Homeopathy and Regular Medicine. Reply by E. V. Stoddard, M. D. 53
On the Action of Hydrate of Chloral. L. A. Harcourt, M. D. 13
Primary Board to Examine Young Men before entering the Study of Medicine 230
Quackery and the National System of Medical Education. H. R. Hopkins, M. D. 50

Cotes, L. B., M. D., Cynanche Trachealis 1
Cundurango 60
" Answers to Correspondents. 73
" At Less Price 116
" By its Friends 362
" How to treat Humbugs 108
" The True and False 140
Cynanche Trachealis and Crural Phlebitis By L. B. Cotes, M. D. 1

D

Dangerous and Fatal result from the use of Hydrate of Chloral 57
Darwinism in Reference to the Eyes of Animals 434
Death of O. C. Gibbs, M. D. 40
" " Thomas Hawkes Tanner, M. D. 79
" " Zina Pitcher, M. D. 362
" " Prof Charles A. Lee, M. D., resolutions on 311
" " Charles A. Weston, M. D. 398
Deaths from Chloral Hydrate 65
Diptheritic Croup, and its Treatment. By F. W. Bartlett, M. D. 321
Diseases of Children, Prize Essay on 116
Doctors, The future state of 79
Dolson J. S., M. D., A Case of Ovariotomy 227
Drunkards, The Treatment of 13
Dunglison, Prof. A new work by the late 273
Dysmenorhœa and Sterility, A successful method of treating certain cases of 79

E

Enucleation in Ovariotomy 35
Ex opthalmic Goitre, or Grave's Disease By J. W. Southworth, M. D. 293
External Iliac, Ligature of. By S. A. Freeman, A. A. Surgeon U. S. A. 291
Extra Uterine Fœtation, Case of. By Harvey Jewett, M. D. 287

F

Flint, Austin, M. D. Pulmonary Phthisis 20
" " " Diagnostic and Prognostic Value of Heat Sounds 54
Fœtus in Utero, Remarkable Presentation of 148
Fracture of the Skull, Case of. By Chas. W. Saunders, M. D. 268

G

Gay, C. C. F., M. D. Hospital Notes 47, 124, 207
" " " On the Employment of Taxis in Strangulated Hernia 256
" " " Operation for Procidentia Uteri 7
" " " Report of a Case of Aneurismal Tumor following penetrating Wound of Shoulder 424
Gibbs, O. C., Death of 40

H

Harcourt, L. A., M. D., Case of Protracted Labor 4
" " " Action of Hydrate of Chloral 13
Haynes, F. L., M. D. The Hypodermic use of Sulphate of Quinia 68
Heart Sounds, Diagnostic and Prognostic Value of. By Austin Flint, M. D. 54
Homeopathy and Regular Medicine, Remark 74
Hopkins, H. R., M. D., Homeopathy and Regular Medicine 15
" " " Quackery and the National System of Medicine Education 50
Hospital Notes. By C. C. F. Gay, M. D. 47, 124, 207
Hutchinson, F. W., M. D. Case of Placenta Prævia 373
Hydrate of Chloral, Dangerous and Fatal Result from the use of 57
" " " Death from 65
" " " Dose of 66

I

In an Obstetrical Case should a physician take his Forceps with him? 71
Indian Cholera. Statistics 31
Intraocular Serous Membrane, and The Nature of Glaucoma. By Dr A. Sichel Jr. Translated by F. W. Abbott, M. D. 161
Inversion of the Uterus, Report of two Cases with Remarks and a Description of the Uterine Repositor By Prof. Jas. P. White, M. D. 383
Iodized Milk 303

L

Labor, Case of Protracted, By L. A. Harcourt, M. D. 4
Laceration of the Perineum, the best period of operating for 70
Lee, Prof Charles A., M. D., Resolutions on the Death of 371
Ligature on the External Iliac, By S. A. Freeman, A. A. Surgeon U. S. A. 291
Liver, Action of Mercury on the 63

M

Meat Extract, The Value of 145
McElroy, Z. C., M. D. Clinical Experiences in Private Practice 365

Medical Association, American, an Exhibition for 312
" " " 238, 361, 398
Medical College, Bowdoin 199
" " Buffalo Introductory Lecture in 117
" " " Allumni Association 153
" " " Annual Commencement of 271
" " Detroit 158
" " Rush 153, 273
" Colleges, Commencement of Lecture Terms in 115
" " and Graduates in the U. S 193
" " In Chicago 18[illegible]
" Department Harvard University 289
" Journal, New 273
" " The National 274
" " Vermont 274
" Men Relief for 191
" Preparations 313
" Society, Erie County Annual Meeting. Reception of a portrait of Dr Levi J. Ham 253
" " Genesee County 231
" " NewYork State Meeting of 239
" " " " " " 169
" " Niagara County, Old and New 438
" " West Virginia 443
Medical Students, The Cuban Murder of 19
Mercury, The action of in Children 145
" " " " " On the Liver 63
Miner, Prof. J. F. Clinical Remarks on Rhinoplasty Talicotian Operation. Reported by W. W. Miner, M. D 41
" " Clinical Remarks on Surgical Cases in the Buffalo Hospital of the Sisters of Charity. Reported by F E L. Brecht, 99, 100, 101, 127, 1[illegible]8, 129 177, 179, 180
Miner, W. W. M. D. Appointment of 75
" " " Report of Clinical Remarks on Rhinoplasty, By Prof. J. F Miner 41
" " " Thesis. — Excisions involving the Joints of the upper Extremity. Reports of illustrative Cases 81
Montgomery, H F., M. D. Suits for Mal-Practice. Presidents Address to the Monroe County Medical Society 445
Moore, Prof. E. M., M. D. Two Cases illustrating the production of Vowel and Consonant Sounds 428

### N

New Books 442
New York Academy of Medicine, Resolutions of 142
New York Observer 159
Notice 158

### O

On Cold Bathing in Typhoid Fever 32
On the Application of Carbolic Acid as a local Anæsthetic 63
On the Employment of Taxis in Strangulated Hernia. By C. C. F. Gay, M. D 256
On the Use of Opium, Bromide of Potassium and Cannabis Indicum in Insanity 26
Ovaritotomy, a Case of, By J. S. Dolson, M D 227
Ovariotomy by Enucleation without Clamp, Ligature or Cautery, and without Hemorrhage 35
Ovariotomy Looking after our Laurels 437
Periodical Headache, A new method of Curative Treatment. By F. Bradnack, M D 220
Pitcher, Zina, M D, Death of 362
Placenta Prævia, Case of, Death on thirteenth day after delivery By F. W. Hutchinson, M D 373
Pneumonia, Treatment of 150
Post Mortem Examinations, short notes on 131
Post Mortem Poisoning 75
Potassium, Bromide, Substituted for Iodide 152
Primary Board, appointed to examine Young Men before entering the Study of Medicine 230
Prize Essays: American Medical Editors' Association 434
Procidentia Uteri. By C. C F. Gay, M D. 7
Promotion, J. H Baxter, M. D. 311
Prostate Gland, Pathology of the 150
Pulmonary Phthisis, By Austin Flint, M. D 20
Pulse, Varieties of the 102

### Q

Quinine, A new and important Property of 139
" Intemperance in Use of 187

### R

Relapsing fever in Carlisle 149
Removal of a large Fibro-Cystic Tumor of the Uterus by Gastrotomy, by Prof. T. Gaillard Thomas, M. D 436
Respirator, an Oakum 130
REVIEWS.—Insanity. Its Dependence on Physical Disease, by John P. Gray, M. D. 36
Byford on the Uterus 37
The Physiological action and Therapeutic Use of Chloral, by J. C. Andrews, M. D 37
Amputation of Redundant Scrotum in the treatment of Varicocele, by M H Henry, M. D 37
Modern Operation for Cataract, by Hasket Derby, M. D 38
Bossanger's Catalogue of Anatomy 38
The Student's Chart of the Sympathetic Nerve, by Ralph M. Townsend, M. D 38
Twenty eighth Annual Report of the Managers of the State Lunatic Asylum 38
Health Officers' Annual Report of the City of Rochester, by Harvey F. Montgomery, M. D. 39
On Syphilitic Epilepsy, by Reuben A. Vance, M D. 76
Plastics and Orthopedics, by David Prince, M. D 76
The late Dr. Conolly, of Hanwell, England, by Charles A Lee, M. D. 76
Artificial Induction of Labor in Uræmia, by Samuel C. Busey, M. D. 76
Minutes of the 22d Annual Meeting of the American Medical Association 77
Transaction of the Minnesota State Medical Society 77
A Treatise on the Nervous System, by William A Hammond, M. D. 77
Opium and Opium Appetite, by Alonzo Calkins, M. D. 78

Tanner on Infancy and Childhood, by Meadows ........................... 78
The Physician's Prescription Book.. 117
Hand Book of Practical Obstetrics, including Anæsthesia, by John Tanner, M. D. ........................... 117
Essay on growths in the Larynx, by Morell Mackenzie, M. D. ........... 117
The Physical Diagnosis of Brain Disease. by Reuben A. Vance, M. D... 118
Electrolysis and its Application to treatment of Disease, by A. D. Rockwell, A. M., M. D... ........... 154
Contributions to Practical Laryngoscopy, by A. Ruppaner, A. M., M. D. 155
Introductory Lecture to the Course on Pathological Anatomy at the University of Philadelphia, by Joseph G. Richardson, M. D. ........... 155
The First Annual Report of the Board of the Free Medical and Surgical Dispensing Association of Buffalo. 155
Practical Therapeutics, by Edward John Warring, M. D. ............... 155
Prolapsus Uteri, by Thomas Addis Emmett, M. D. ..................... 157
The Management of Infancy, Physiological and Moral, by Andrew Combe, M. D. ..................... 157
Retrospect of Uterine Pathology and Therapeutics in the United States, Henry Miller, M. D. ............... 157
Anatomical Remembrancer, or Complete Pocket Anatomist, by C. E. Isaacs, M. D. ..................... 157
Handy-Book of the Treatment of Women's and Children's Diseases, by Emil Dillnberger ............... 158
Essentials of the Principles and Practice of Medicine, by Henry Hartshorne, M. D ..................... 158
Bright on Cancer, its Classification and Remedies ..................... 193
Thoughts on Chronic Inversion of the Uterus, by H. Miller, M. D. ........... 194
Remarks on Wine and Alcohol, by Charles A. Lee, M. D. ............... 195
The Prevention of Abcesses in Hypodermic Medication, by Reuben A. Vance, M. D. ..................... 195
Clinical Examination of Urine, by Reuben A. Vance, M. D... ........... 195
A Contribution to the Treatment of Vetsions and Flexions of the Unimpregnated Uterus, by Ephraim Cutter, M. D. ..................... 195
The Physician's Dose and Symptom Book, by Joseph Wythes, M. D..... 196
Gems of Pen Art, by Charles B. Knowlton ..................... 196
The Scientific American ............... 196
Skin Diseases, their Description, Pathology, Diagnosis and Treatment, by Tilbury Fox, M. D., Edited by M. H Henry, M. D. ............... 196
Manual of Midwifery, by Alfred Meadows. M. D ............... 197
The Clinical Thermometer, by Z. C. McElroy, M. D. ..................... 197
Physicians' Visiting List for 1872, by Lindsay & Blakiston. ............... 197
Physicians' Daily Pocket Record, by S. W. Butler, M. D. ............... 197
Report to the Surgeon-General on an Improved Method of Photographing Histological Preparations by Sunlight, by Assistant-Surgeon J. J. Woodward, U. S. A. ............... 198
American Practitioner ............... 198
Practical Treatise on Fractures and Dislocations, by Frank Hastings Hamilton, A. M., M. D., LL. D..... 235
A Text Book of Pathological Histology, an Introduction to the Study of Pathological Anatomy, by Dr. Edward Rindfleisch, Translated from the German by William C. Kloman, M. D., and E. T. Miles, M. D. ..... 236
Emergencies and how to treat them, by Jos. N. Howe, M. D. ........... 236
The Druggist's General Receipt Book, with Veterinary Formulary, by Henry Beasley ..................... 237
Disorders of the Nervous System in Childhood, by Chas. West, M. D.... 237
Neumann's Hand Book of Skin Diseases, translated from the German by Lucius D. Buckley, M. D. ...... 237
Functions and Disorders of the Reproductive Organs in Childhood, Youth, Adult Age and Advanced Life, by William Acton, M. R. C. S. 237
Restorative Medicine, by Thos. King Chambers, M. D. ............... 238
The Principles and Practice of Surgery, by John Ashhurst, M. D. Review by C. H Richmond, M. D., Syracuse, N. Y. ............... 274
A Clinical Manual of Diseases of the Ear, by Lawrence Turnbull, M. D.. 280
Fireside Science. A Series of popular and scientific Essays upon subjects connected with every-day Life, by James R. Nichols, A. M, M. D..... 313
War Department. Surgeons General's Office Circular, No. 3, Report of Surgical Cases in the Army, from 1855 to 1871, by Geo A Otis, M. D. 313
Proceedings of the Royal Society.... 314
Transaction of the Twenty-Sixth Annual Meeting of the Ohio State Medical Society ..................... 314
Headaches, their Causes and their Cure, by Henry G. Wright, M. D. etc ..................... 314
On the Treatment of Pulmonary Consumption. by James Henry Bennet, M, D ..................... 315
Transaction of the American Medical Association ..................... 315
Neuralgea and the Diseases that resemble it. By Francis E. Anstie, M. D. ..................... 315
A Practical Treatise on Bright's Diseases of the Kidneys, by T. Grainger Stewart, M. D., etc ...... 316
Transactions of the Medical Society of the State of Pennsylvania. 316
Modern Medical Therapeutics, by G. H. Napheys, A M, M. D ...... 317
Medical Thermometry and Human Temperature, by C. A Wunderlich M. D. Trans. by Edw. Seguin, M. D. 317
A Hand-Book of Therapeutics, by Sidney Ringer, M. D., etc ........ 317
Boylston Prize Essay, 1871, by B. Joy Jeffries A. M, M. D.... ..... 318
The Works of Sir James Y. Simpson, Bart., M. D., D. C. L. Volume II. Edited by Sir W. G. Simpson, Bart. B. A ..................... 318
Diagrams, by J M. Toner, M. D ..... 318
A Treatise on Human Physiology; designed for the use of students and practitioners of medicine, by John C. Dalton, M.D. Fifth Edition 363

Physiology of the Soul and Instinct, as distinguished from Materialism, by Martin Paine, A. M , M. D , LL.D. 363
Can Chloroform be used to facilitate Robbery? by Stephen Rogers, M.D 399
Transactions of the American Opthalmological Society 399
Eating and Drinking, by George M Beard, M. D 399
Stimulants and Narcotics, by George M. Beard, M, D 399
Plain Talk about Insanity, by T. W. Fisher, M. D 400
Animals and Vegetable Parasites of the Human Skin and Hair, by B. Joy Jeffries, A. M., M. D 400
An Introduction to Pathology and Morbid Anatomy, by T. Henry Green, M. D 400
Pulmonary Consumptions, by C. B. J Williams, M D., F R. S., & Chas. T. Williams, M. A., M. D 401
Injuries to Nerves and their consequences, by S. Weir Mitchell, M.D. 4 1
Lectures on Aural Catarrh; or the commonest forms of Deafness and their cure, by Peter Allen M. D 438
A Treatise of Diseases of the Bones, by Thomas M. Markoe, M. D 439
Earth as a Topical application in Surgery by Addinell Hewson, M. D 439
History of edicine from the earliest ages to the commencement of the Nineteenth Century, by Robley Dunglison, M. D., LL D. Edited by Richard J. Dunglison, M. D 440
Dr. Ri by's Obstetric Memoranda, by Alfred Meadows, M. D 440
Memoranda on Poisons, by the late Thomas Hawkes Tanner, M. D., F. L. S 440
Parturition without Pain, by M. L. Holbrook. M. D 441
Diseases on Women, by T. Gaillard Thomas, M. D 441
A Treatise on Diseases of Infancy and Childhood, by J. Lewis Smith, M. D. 482
The Urine and its Derangements, by George Harley, M. D. 482
The Treatment of Venereal Diseases a Monograph on the method pursued in the Vienna Hospital, by M. H. Henry, M D. 483
Clinical Lectures on the Diseases of Women, by Sir James Y. Simpson, Bart., M. D., D. C. L 483
Rhinoplasty, Talicotian Operation, Result illustrated, Clinical lecture by Prof. J. F. Miner, M. D , Reported by W. W. Miner, M. D. 41
Richmond, C. H., M. D., Clinics in Bellevue 112
Rider, Charles E., M D . Abstract of remarks on Diseases of the Eye and Ear, and their treatment 89
Rochester, Prof. Thomas F., M. D., Clinical Remarks in the Buffalo Hospital of the Sisters of Charity, Reported by Edward N. Brush 121, 264, 266

### S

Salivation, fatal, from Bichloride of Mercury 151
Saunders, Charles W , M. D , Case of Fracture of the Skull 268
Scarlet Fever Poison, Source of 66
Should a Physician take his Forceps with him in an Obstetrical Case? 71
Skin Grafting, an instrument to facilitate 308
Skin Diseases, The Student's Classification of, By J W. Southworth, M. D. 119
Small Pox, Disinfectant, Treatment of 304
" Treatment of 305
Southworth, J. W , M. D., Abcess of Temporal Region from Gunshot Wound Extraction of Ball 229
" Apyretic Malarial Menorrhagia 174
" Ex opthalmic Goitre 293
" The Student's Classification of Skin Diseases 119
Standard of Medical Education 33
Steam Atomizer, a new 396
St Louis Medical Journal 239
Stoddard, E. V., M D., Reply to Homeopathy and Regular Medicine 53
Strangulated Hernia, a plan for reduction by Taxis. 306
Successful method of treating certain cases of Dysmenorrhœa and Sterility 79
Suits for Malpractice, President's Address to the Monroe County Medical Society 445
Sulphate of Quinia, the Hypodermic use of, by F. L. Haynes, M. D. 68
Surgeon-General U. S. A., Report of the 199
Syphilis, the treatment of 105

### T

Tanner, Thomas Hawkes, M. D., Death of 79
The Periodicals 442
Theory of Vision, On the recent advances in the, by H. Helmholtz, Translated by F.W. Abbott, M. D 209, 245, 295, 346, 376, 416, 474
Thesis—Excisions involving the joints of the upper extremity, Reports of illustrative cases, by W W. Miner, M D. 81
Thomas, Prof T Gaillard, M. D., Removal of a large Fibro-Cystic Tumor of the Uterus by Gastrotomy 466
Tobacco 304
Toner's Medical Register and Directory 404
To the Medical Profession in the United States 189
Transfusion of Blood, Arterial 232
Treatment of Capillary Bronchitis in Children by Warm Vapor 19
Two cases illustrating the production of Vowel and Consonant sounds, by Prof. E. M. Moore, M D 428
Typhoid Fever, Cold Bathing in 30

### U

Ulcers of the Cervix Uteri 67
Undulatory Theory applied to Sensation 151
Urea, The formation of 67

### V

Vaccination in Small Pox 234
Varicose Veins, New operation for the cure of 307

### W

Wanted 198
Wanted, A Rara Avis 62
Weston, Charles A., M. D. Death of 398
Where shall we send our Consumptive Patients 144
White, Prof. James P., M. D. Report of Two Cases of Inversion of the Uterus with Remarks and a Description of the Uterine Repositor 383

### Books and Pamphlets Received.

---

A Physicians Counsels to Woman, in Health and Disease. By Walter C. Taylor, A. M., M. D. Springfield: W. J. Holland & Co., 1871.

School Material. J. W. Schermerhorn & Co., Publishers and Manufacturers, New York. A complete illustrated catalogue of the latest improved material demanded by the schools.

Comments of the medical press on the alleged malpractice suit of Walsh vs. Sayre. New York: Shaw & Co., 1871.

The Rejected Address. Man's True Relation to Nature; his Origin Character and destfny. By T. P. Wilson, M. D., Editor Ohio Medical and Surgical Journal. Cleveland: Robinson & Co., 1871.

Minutes of the Twenty-Second Annual Meeting of the American Medical Association, held in the City of San Francisco, May 2d, 3d, 4th, 5th, 1871.

Reply to the attack of Dr. E. S. Gaillard, by D. W. Yandell, M. D. Louisville: Morton & Co., 1871.

Artificial Induction of Labor in Uræmia. By Samuel C. Busey, M. D.

On Syphilitic Epilepsy. By Reuben A. Vance, M. D. New York: F. W. Christern, 1871.

